# Survey of Spinal Cord Stimulation Hardware Currently Available for the Treatment of Chronic Pain in the United States

**DOI:** 10.3389/fpain.2020.572907

**Published:** 2020-11-23

**Authors:** Josephine A. Clingan, Ashish Patel, Dermot P. Maher

**Affiliations:** ^1^Southeastern Interventional Pain Associates, Atlanta, GA, United States; ^2^Geisinger Medical Group, Danville, PA, United States; ^3^Johns Hopkins School of Medicine, Baltimore, MD, United States

**Keywords:** spinal cord stimulation, neuromodulation, chronic pain, practice management, pain treatment, medical devices

## Abstract

**Background:** The number of spinal cord stimulator (SCS) units sold in the United States (US) for the treatment of chronic pain has increased with a corresponding expansion in the number of different SCS platforms available. Each marketed stimulator has several unique features, indications, and limitations, which distinguish one from the other and makes the selection of appropriate hardware possible for optimal patient care. There are an even greater number of similar and overlapping features between SCS.

**Measures:** We used market analysis techniques to survey the currently available SCS technology. We then reviewed published device specifications and manuals for comparison of features.

**Outcomes:** As of 2020, there are nine commonly used SCS platforms made by four manufacturers including four SCS units from Abbott, three from Boston Scientific, and one each from Medtronic and Nevro.

**Conclusions:** A working understanding of each SCS product's nuances is needed for selecting the most appropriate device with which to manage chronic pain patients. Here we present a brief survey of currently available SCS hardware in the US and the features that make each product unique.

## Introduction

The point prevalence of chronic low back pain (cLBP) among all adults in the United States (US) is 13.1% (1). Several factors have been identified to confer a more than doubling of the adjusted odds ratio (aOR) of cLBP including being between 50 and 69 years old (aOR 2.03–2.07), having less than a high school education (aOR 2.27), having an annual household income < $20,000 (aOR 2.29), income derived primarily from disability (aOR 2.62), depression (aOR 3.30–10.62 depending on severity), sleep disturbances (aOR 3.90), and other medical comorbidities (aOR 2.49–6.09) (1). The lifetime prevalence of acute LBP is nearly 80% in the United States (2). There is concern that as the US population ages and attains increasing risk factors for the development of cLBP, there will be a need for increased treatments (3).

The treatment of cLBP pain represents a major financial burden on the US health-care system. The 12 month health-care expenditures of adult patients with LBP in the US was found to be $25,613 (95% confidence interval $25,569–$25,657) among patients who underwent spine surgery compared to $795 ($790–800) among patients who chose non-surgical treatments (4). The two major considerations when choosing a spinal cord stimulation (SCS) system are efficacy, which is often equivalent to spine surgery, and cost, which is substantially less than spine surgery. SCS represents a continuously evolving technology with evidence for cost-effective management of cLBP. The use of older, non-rechargeable implanted pulse generators (IPGs) was associated with similar incremental cost utilization ratio (ICUR) compared to surgical reoperation for the treatment of LBP (0.59 vs. 0.83) (5). The use of SCS for the treatment of neuropathic leg and LBP was associated with higher upfront costs compared to conventional medical therapy ($19,486 vs. $3,994) but increases in health-care-related quality of life and EuroQol-5D (EQ-5D) scores at 6 months (6).

The utilization of SCS therapy for the treatment of chronic painful conditions continues in the US due to well-documented efficacy. The rapid development of SCS systems over time necessitate continuously updated reviews of available hardware (7). There exists a number of different products available in the US, each with its own unique features, indications, and limitations. The purpose of this review is to succinctly present the unique and differentiating aspects of commonly available SCS systems currently available on the US market. The intention of the review is that it will be a periodically updated resource that will reflect changes in available SCS products.

## Methods

This study only gathered data that was publicly available. As such, the study did not require Internal Review Board approval. Internet search tools including MEDLINE, EMBASE, Google scholar, and Google were used to identify SCS products. Searches included terms such as “spinal cord stimulation” “dorsal column stimulation.” Title and abstracts were iteratively reviewed for relevance with particular emphasis placed on high-quality health-care market assessments and product details provided by either device manufactures or independent, non-biased sources (e.g., FDA and other government agencies). Data was excluded if it described products that were not approved and available for patients to use in the United States in 2020 for the intended implantation in spine. This resulted in the exclusion of SCS devices available in other countries as well as for other indications, such as vagal nerve stimulators, which were not relevant to our analysis. Additionally, reviews focusing on mechanism of action or clinical effectiveness were not included as this was not the primary goal of the manuscript. All authors were involved in gathering and interpreting information. Unique features of products were then confirmed using from several sources, including product manuals, medical conference proceedings, published investor and business development reports, publicly available company due diligence reports, peer-reviewed medical literature, and device manufacturer-produced literature. When necessary, clarification was made through requesting additional documentation from device manufacturer sales teams and engineering support personnel. Endnote X9 was used to manage references and data sources (Clarivate Analytics, Philadelphia PA).

## Results

There are currently nine different SCS units commonly-available for the treatment of pain in the United States. The features of each device are presented in Table 1. Data was derived from a number of sources (8–16). Eight of the product's leads are intended to be placed over the dorsal columns of the spinal cord, and one product's leads are intended to be placed over the dorsal root ganglion. While the dorsal root ganglion is not technically a part of the spinal cord, the provided mechanism of action and treatment indications of this device makes it more appropriately discussed with spinal cord stimulators rather than peripheral nerve stimulators. Different batteries have unique warranty of between 2 and 10 years, while most are expected to last longer than this prior to the need to be replaced. Four of the systems do not use rechargeable batteries and five of the systems do use rechargeable batteries. Recharging times range from 15 to 120 min. The frequency and rate of recharging is generally a function of the stimulation settings. With regards to MRI compatibility, five of the spinal cord stimulator systems are full-body conditional, and two are compatible with only head and extremity imaging, one is not MRI compatible, one is compatible only with cranial imaging. Each device has a unique definition of conditionality with MRI that should be carefully considered prior to imaging. Two of the devices do not need to be deactivated while driving, and seven do need to be deactivated while driving when used to treat LBP and/or lower extremity pain. Seven are capable of burst frequency programing. The exact definition of “burst frequency programming” varies between devices and is provided in the footnotes of Table 1. The sizes of the IPG for each system are presented in Table 2. The Medtronic Intellis system currently has the thinnest IPG. Older units that still appear on company websites but are not highly marketed are listed in Table 3.

**Table 1:** Features of currently available spinal cord stimulation systems.

**Manufacturer**	**Device**	**Date of FDA approval**	**Upgradeable software**	Battery life*	**Rechargeable battery**	**Recharging frequency**	**MRI compatibility**	**Turn off while driving**	**Turn off while sleeping**	**Burst capable**	**Unique factors**	**Other**
Boston Scientific	WaveWriter	January 2018	No	Five year warranty, usually lasts 12 years	Yes	15–30 min daily	Head only	Yes	At patient's discretion	Yes	1. Paresthesia-free stimulation at 1.2 kHz 2. Paresthesia-free “micro-burst” programing 3. Can run both burst and tonic stimulation simultaneously	Currently involved in litigation with Nevro over patent laws concerning frequency
	Precision Montage	May 2016	No	Five year warranty	Yes	120 min every 2–3 days	Full body conditional	Yes	At patient's discretion	Yes		
	Precision Novi	June 2015	No	Two year warranty, usually lasts 5 years	No		No	Yes	At patient's discretion	Yes	1. Capable of burst or 1.2 KHz stimulation but not recommended as it will decrease battery life 2. Cannot do burst and 1.2 KHz simultaneously	
Medtronic	Intellis	July 2017	Yes	Nine year warranty	Yes	60 min every 1–5 days	Full body conditional	Yes	At patient's discretion	No	1. Can use “low dose” 40 Hz” or “high dose” 1000 Hz stimulation	1. Purchased Stimgenics in January 2020 for undisclosed amount. Conducting RCT for incorporation of proprietary waveform that targets glial cells2. Smallest battery
Nevro	Senza Omnia	November 2019	Yes	Minimum 10 year	Yes	45 min daily	Full body conditional	No	No	Yes	1. Does not require mapping 2. can simultaneously run burst with high frequency (10 kHz) or lower frequency	
Abbott	Proclaim XR Recharge-Free	September 2019	Yes	Five year warranty	No	NA	Full body conditional	Yes	At patient's discretion	Yes	1. No need to recharge 2. Can be controlled through Apple device, such as iphone, with Bluetooth connection 3. Postural changes affect stimulation intensity	
	Proclaim Elite with burst	October 2016	yes	Up to 10 years	No	NA	Full body conditional	Yes	At patient's discretion	Yes	1. No need to recharge 2. Can be controlled through Apple device, such as iphone, with Bluetooth connection 3. Postural changes affect stimulation intensity	
	Prodigy MRI IPG	October 2016	Yes	Ten year warranty	Yes	45 min 1–3 times per week	Head and extremity only	Yes	At patient's discretion	Yes	1. Can be controlled through Apple device, such as iphone, with Bluetooth connection 2. Postural changes affect stimulation intensity	
	Proclaim DRG Neurostim	November 2016	Yes	5 to 6 years on average	No	NA	Head and extremity conditional	No	At patient's discretion	No	1. Only DRG stimulation product currently available 2. Can be controlled through Apple device, such as iphone, with Bluetooth connection	

*DRG, dorsal root ganglion; FDA, Food and Drug Administration; Hz, hertz; IPG, implantable pulse generator; KHz, kilohertz; MRI, magnetic resonance imaging; RCT, randomized controlled trial*.

* *Battery life and frequency of charging is technically a byproduct of individual patient usage. Higher usage will result in more frequent charging sessions and decreased battery life. A patient's recharge routine may vary depending on your stimulation parameters. High power users will require more frequent charging*.

*Boston Scientific Definition of Burst: 2–6 bursts at 450 Hz frequency*.

*Boston Scientific Definition of Microbursts: between 0 and 1 s range. Amplitude is set at 50% of the patient perception threshold. Pulse width is 210 ms, rate is 450 Hz intraburst with 40 Hz interburst. Microburst is 6 pulses on 12. Ms, followed by 12.5 ms off*.

*Nevro Definition of Burst: 2–4 bursts, at 500 Hz*.

*Abbott Definition of Burst: 40, 500 Hz of 5 s spikes; with cycles of (1) 30 s on, 30 s off, (2) 30 s on, 3 min off, (3) 30 s on, 6 min off*.

**Table 2:** Size comparison of implantable pulse generators.

**Manufacturer**	**Device**	**Size (depth × height × length) mm**
Boston Scientific	Precision Plus	10 × 54 × 45
	Precision Novi	11.3 × 70.9 × 49.5
Medtronic	Intellis	6 × 57 × 47
Nevro	Senza II	10 × 56 × 46
	Omnia	10 × 56 × 46
Abbott/ St. Jude	Eon Mini	9 × 50 × 57
	Prodigy MRI	9 × 48 × 53
	Proclaim Elite	13 × 56 × 50
	Proclaim XR	13 × 56 × 50
	Proclaim DRG	13 × 61 × 50

*mm, millimeter*.

**Table 3:** Older products not discussed but still appear on company product websites.

**Name**	**FDA approval date**
Boston Scientific Precision	2004
Boston Scientific Precision Plus	2005
Boston Scientific Precision Spectra	2013
Medtronic Restore Advanced	7/2006
Medtronic Restore Ultra	2/2008
Medtronic Restore Sensor	11/2011
Medtronic Prima Advanced Surescan MRI	2013
Nevro Senza	2015
Nevro Senza II	2018

## Conclusions

The ongoing development of SCS technology has led to the commercialization of several products on the US market, each with unique properties. This ever-expanding armamentarium allows physicians to individualize pain treatment and overcome previously existing treatment barriers. The current selection of SCS technologies has improved over previous generations through the refinement of SCS technologies including the miniaturization of IPGs, extended battery life, unique/novel waveforms and programming options, improved designs to ease trials and implantation, and a reduction in limitations of use, such as the expansion of MRI compatibility. We anticipate that this market will continue to be develop.

## Discussion

The technology for SCS is continuously improved with the goals of refining current treatment applications and expanding therapeutic indications. In 2019, there was a decrease in the US SCS market overall. However, by 2025 the US SCS market is expected to increase by 5–10% compounded annual growth (17).

The most common new trend is the development of multiple waveform-capable product lines and individual products, such as the non-rechargeable Abbott Proclaim (burst and traditional) and the Nevro Omnia (burst, traditional, and high frequency). The optimal waveform and programming for the treatment of different painful phenotypes is currently being investigated in several ongoing clinical trials with results expected in 2022 or later (NCT03681262, NCT03957395, and NCT03014583). Currently there is a paucity of evidence from direct comparison of different waveforms in pragmatic clinical trial settings to adequately inform healthcare decisions.

The development of future SCS technology, including novel platforms and programming, will continue to occur in order to satisfy ongoing and unmet patient needs. Predictions of any new technology remains would be vague for two reasons. First, any new SCS technology would need to be formally evaluated in clinical trials for both safety and effectiveness prior to commercialization. Second, the need for protections of novel intellectual property makes very little information available to the public. Future iterations of this or similar manuscripts will strive to provide details of such new and emerging technology. SCS leads are a crucial component of an implantable SCS system. The use of different numbers and types of leads (paddles vs. percutaneous, one lead or two) can result in significant changes in the clinical profile of many SCS systems and is an additional important consideration for implanting physicians to consider.

The evidence on SCS for the treatment of pain is expanding. While the focus of this manuscript was to survey the characteristics of the hardware, unique clinical outcomes and head-to-head comparisons are extremely important considerations. The currently published reviews of SCS clinical utility do not allow for several practical questions to be answered such as the ability to decrease opioid use or increase in functional capacity. There is also a dearth of large-scale and long-term data regarding the utilization of high-cost health-care resources after implantation of a spinal cord stimulator, such as the avoidance of spine surgery. With the increased utilization of SCS to treat LBP in non-previously operated spines, additional data will be needed to delineate the most effective SCS treatment algorithms in these patients. Physicians who use SCS to treat pain are now faced with several options in the US market with both unique and overlapping features.

## Data Availability Statement

The raw data supporting the conclusions of this article will be made available by the authors, without undue reservation.

## Author Contributions

All authors contributed equally and meet the criteria for authorship based on International Committee of Medical Journal Editors.

## Conflict of Interest

The authors declare that the research was conducted in the absence of any commercial or financial relationships that could be construed as a potential conflict of interest.
